# MANF/EWSR1/ANXA6 pathway might as the bridge between hypolipidemia and major depressive disorder

**DOI:** 10.1038/s41398-022-02287-0

**Published:** 2022-12-30

**Authors:** Ke Xu, Peng Zheng, Shuang Zhao, Mingyang Wang, Dianji Tu, Qiang Wei, Jinzhou Feng, Haiyang Wang, Jianjun Chen, Peng Xie

**Affiliations:** 1 Department of Neurology, The First Affiliated Hospital of Chongqing Medical University, Chongqing, China; 2 National Health Commission Key Laboratory of Diagnosis and Treatment on Brain Functional Diseases, The First Affiliated Hospital of Chongqing Medical University, Chongqing, China; 3 Department of Pathophysiology, Chongqing Medical University, Chongqing, China; 4 Kunming Institute of Zoology, Chinese Academy of Sciences, Kunming, China; 5 Department of Clinical Laboratory, Xinqiao Hospital, Third Military Medical University, Chongqing, China; 6 Department of Laboratory Medicine, The First Affiliated Hospital of Chongqing Medical University, Chongqing, China; 7 Key Laboratory of Psychoseomadsy, Stomatological Hospital of Chongqing Medical University, Chongqing, China; 8 Institute of Life Sciences, Chongqing Medical University, Chongqing, China

**Keywords:** Depression, Diagnostic markers

## Abstract

Major depressive disorder (MDD) involves changes in lipid metabolism, but previous findings are contradictory. Mesencephalic astrocyte-derived neurotrophic factor (MANF) is considered to be a regulator of lipid metabolism. To date, the function of MANF has been studied in many brain disorders, but not in MDD. Therefore, to better understand the role of lipids in MDD, this study was conducted to examine lipid levels in the serum of MDD patients and to investigate the potential function of MANF in MDD. First, the data on total cholesterol (TC), low-density lipoprotein cholesterol (LDL-C), high-density lipoprotein cholesterol (HDL-C), and triglyceride (TG) in serum from 354 MDD patients and 360 healthy controls (HCs) were collected and analyzed. The results showed that there were significantly lower concentrations of TC and LDL-C in MDD patients compared with HCs, and TC levels were positively correlated with LDL-C levels. Bioinformatics analysis indicated that MANF/EWSR1/ANXA6 pathway might serve as the connecting bridge through which hypolipidemia played a functional role in MDD. Second, to verify this hypothesis, serum samples were collected from 143 MDD patients, and 67 HCs to measure the levels of MANF, EWSR1, and ANXA6 using ELISA kits. The results showed that compared to HCs, MDD patients had a significantly lower level of MANF and higher levels of ANXA6 and EWSR1, and these molecules were significantly correlated with both TC level and Hamilton Depression Rating Scales (HDRS) score. In addition, a discriminative model consisting of MANF, EWSR1, and ANXA6 was identified. This model was capable of distinguishing MDD subjects from HCs, yielded an area under curve of 0.9994 in the training set and 0.9569 in the testing set. Taken together, our results suggested that MANF/EWSR1/ANXA6 pathway might act as the bridge between hypolipidemia and MDD, and these molecules held promise as potential biomarkers for MDD.

## Introduction

Major depressive disorder (MDD) is a major mental disorder with high mortality and disability [1, 2]. The most common pathophysiological explanation of MDD relies on the monoamine hypothesis, which indicates that depressive symptoms relate to a lack of neurotransmitters such as serotonin [3]. As of now, selective serotonin reuptake inhibitors and tricyclic antidepressants are available as therapeutic options for MDD, while some MDD patients still fail to benefit from these antidepressants. In recent years, an increasing number of studies have suggested that lipid abnormalities may play an important role in the pathophysiology of MDD [4–6], but findings regarding this association are inconsistent. Some studies found lower total cholesterol (TC) in subjects with MDD versus control [7, 8], while others reported higher TC [9] or found no differences [10]. These contradictory findings might result from the relatively small sample size, samples with a restricted age range, or single-sex groups in some studies. These confounding factors reduce the comparability of studies and the generalizability of results to clinical practice [11]. Meanwhile, levels of low-density lipoprotein cholesterol (LDL-C), high-density lipoprotein cholesterol (HDL-C), and triglyceride (TG) were also assessed for depression [9], though less extensively. Thus, it is necessary to further assess the associations between serum lipids and MDD in large samples with a broad age range and both men and women.

Nowadays, diagnosis in psychiatry is still based on symptomatology and lacks any molecular foundation, which limits the development of diagnostic measures. Psychiatric diseases are increasingly being studied by using peripheral tissues such as blood from patients [12–14], and some molecular mediators identified from these studies may serve as potential biomarkers [15, 16]. Mesencephalic astrocyte-derived neurotrophic factor (MANF) is a newly discovered secreted neurotrophic factor that contains two distinct domains. The N-terminal domain is a saposin-like protein, which interacts with lipids and membranes [17], and the C-terminal domain has reductase or disulfide isomerase activity [18]. Human blood contains MANF [19], which is expressed by most tissues, especially neurons [20]. Several stress signals can induce MANF and it acts as a cytoprotective factor [18]. Among other functions, MANF is a crucial regulator in lipid metabolism [21]. The concentrations of MANF in the serum are higher in patients with hyperlipidemia [22], and the levels were correlated with TC and LDL-C levels [23]. In addition, studies have shown that MANF could exert protective functions in multiple brain disorders, such as intracerebral hemorrhage and Parkinson’s disease [24, 25].

These findings emphasize the importance of serum lipids in MDD and MANF in regulating lipid metabolism. However, the concentration and role of MANF in the serum of MDD patients have received considerably less attention. Further investigations of MANF in MDD can help us better understand the role of lipids in the onset and development of depression, as the results may provide a valuable network among serum lipids, MANF and MDD. Therefore, the present study was conducted to assess the changes of serum lipid levels in MDD patients and investigate the potential role of lipid metabolism regulator MANF in depression. Furthermore, the correlative relationships of MANF and its related molecules EWSR1 (Ewing sarcoma breakpoint region 1) and ANXA6 (Annexin A6) with clinical characteristics and serum lipid levels were also investigated.

## Materials and methods

### Subject recruitments

The study received approval from the local ethics committee of Chongqing Medical University. Written informed consent was obtained from each participant before blood sampling. In total, 714 subjects (sample set 1: 354 MDD patients and 360 healthy controls (HCs)) were included between November 2021 and June 2022 to assess the serum lipids level, and another 210 subjects (sample set 2: 143 MDD patients and 67 HCs) were enrolled to study the underlying role of serum MANF in depression. The diagnosis of MDD was confirmed by two trained psychiatrists according to the Diagnostic and Statistical Manual of Mental Disorders-version IV criteria, and the International Statistical Classification of Diseases and Related Health Problems criteria, 10th revision. All MDD patients enrolled from the Department of Psychiatry, First Affiliated Hospital of Chongqing Medical University. Considering that antipsychotic medication may affect the levels of serum lipids, MDD patients were divided into drug-naive MDD (DN-MDD) group and drug-treatment MDD (DT-MDD) group. The DN subgroup was defined as first-episode MDD and never received any antidepressant treatment. And the DT subgroup was defined as having only received antidepressant treatment before. HCs were recruited from the Medical Examination Center, First Affiliated Hospital of Chongqing Medical University. Subjects receiving fatty acid supplements, statins, interferon, or cortisone in the previous three months were excluded from all groups. Meanwhile, serum samples used for measuring the MANF, EWSR1, and ANXA6 levels were collected from subjects in sample set 2.

Based on the basic clinical documentation, the depression severity in MDD patients was rated by the 17-item Hamilton Depression Rating Scale (HDRS). Clinical data were extracted from the patient discharge letters. We also collected patient’s information on marital status (single/married/divorced/widowed), levels of education (low/middle/high), work status (unemployed/others not working/working/in training/retired/housewife/-man), drinking status (never/moderate/heavy), and smoking status (never/moderate/heavy). Meanwhile, detailed information on antidepressant use was collected in the DT-MDD subgroup.

### Bioinformatics analysis

The Ingenuity Knowledge Base’s ingenuity pathway analysis (IPA) is an accurate online biomedical analysis tool that helps researchers to predict existing interaction networks between molecules and understand their properties [26]. Here, based on a previous study [27], we used three keywords (MDD, MANF, and cholesterol) and ‘Grow’/‘Path Explorer’ tools to search for potential networks among MDD, MANF, and cholesterol.

### Serum lipids analyses

Blood for lipid analyses was sampled at 7:00 AM after overnight fasting. The samples were transported to the Center for Clinical Molecular Medical detection, First Affiliated Hospital of Chongqing Medical University within one hour. Levels of TC, HDL-C, and LDL-C, as well as TG, were determined on the COBAS C8000 Modular Analyzer (Roche Diagnostics, Mannheim, Germany) according to routine laboratory methods.

### Measurement of MANF, EWSR1, and ANXA6 in serum

Peripheral whole-blood samples were collected from each participant by venous puncture. Consistent with our previous study [28], serum was isolated by centrifugation at room temperature at 3000 × *g* for 15 min, aliquoted, and stored at −80 °C until use. Total MANF, EWSR1, and ANXA6 were measured using commercially available high-sensitivity ELISA kits from MEIMIAN (Jiangsu, China) by two blind experimenters. All ELISA kits were used following the manufacturer’s instructions. The detection limit was 0.25 ng/ml for the MANF assay, 10 pg/ml for the EWSR1 assay, and 1 ng/ml for the ANXA6 assay. In addition, sample collection and storage time did not differ among groups.

### Statistical analysis

Statistical analyses were performed using Statistical Package for the Social Sciences, version 20 (IBM Corp., Armonk, NY, USA). The results were presented as mean ± standard error of the mean (SEM). Comparisons of demographic and clinical data for continuous variables between groups were conducted using independent sample *t*-tests (if the data fit the normal distribution between two groups), Mann–Whitney U test (if the data did not fit the normal distribution between two groups), Kruskal–Wallis test (if the data did not fit the normal distribution between three or more groups), or ANOVA followed by post hoc comparison with the Bonferroni test (if the data fit the normal distribution between three or more groups), where appropriate. The variances between MDD and HCs were calculated using Levene’s test; if the variances were not similar between the two groups, the adjusted *p*-value was used. Comparisons of categorical variables were performed using Chi-square test. Pearson correlation coefficient was used to characterize the correlation between two variables. To identify the potential biomarkers for MDD, the logistic-regression analysis was used here. To assess the diagnostic performance of the panel consisting of these potential biomarkers, the receiver operating characteristic (ROC) curve analysis was used to quantify its ability in discriminating MDD patients from HCs in both training and testing sets. The value of the area under the ROC curve (AUC) was the evaluation index of diagnostic performance: ‘excellent’ (AUC = 0.90–1.00), ‘good’ (AUC = 0.80–0.89), ‘fair’ (AUC = 0.70–0.79), ‘poor’ (AUC = 0.60–0.69), or ‘fail’/no discriminatory capacity (AUC = 0.50–0.59) [29]. Significance was set as *p* values of < 0.05 for group comparisons and correlation analyses.

## Results

### Sociodemographic and clinical data

Table 1 summarized the sociodemographic and clinical data of subjects in sample set 1 and 2. Sample set 1 included 360 HCs and 354 MDD patients (130 DN-MDD and 224 DT-MDD). There was no significant difference in sex (*p* = 0.579) and age (*p* = 0.360) between HCs and MDD groups. Meanwhile, sample set 2 included 67 HCs and 143 MDD patients (75 DN-MDD and 68 DT-MDD). No significant difference in sex (*p* = 0.129) and age (*p* = 0.762) distributions were found between groups. The value of HDRS was significantly higher in MDD group than in HCs group (*p* = 8.17E−15). In addition, in sample set 1 and 2 (Tables S1 and S2), the majority of patients were married, well-educated, have jobs, and never drank or smoked. And the anti-depression drugs presented to DT-MDD participants were primarily first-line, i.e., olanzapine, venlafaxine, and escitalopram.

**Table 1:** Clinical details of recruited subjects in the study.

	Sample set 1			Sample set 2		
Characteristics	HCs	MDD	*p* value	HCs	MDD	*p* value
Sample size (*n*)	360	354	–	67	143	–
Sex (male/female)	124/236	115/239	0.579^a^	29/38	65/78	0.129^a^
*Age (years)*						
Range	23–69	18–70	–	19–66	18–66	–
Mean ± SEM	39.47 ± 0.5	40.08 ± 0.90	0.360^b^	37.21 ± 1.33	37.61 ± 1.14	0.762^b^
HDRS (mean ± SEM)		33.41 ± 0.49	–	3.36 ± 0.32	33.46 ± 0.76	8.17E−15^c^
*Duration of illness (months)*						
Range	–	0.33–360		–	0.33–180	
Mean ± SEM		50.91 ± 3.28		–	39.85 ± 3.60	

Continuous variables are expressed as mean ± standard error of the mean (SEM).

*HCs* healthy controls, *HDRS* Hamilton Depression Rating Scale, *MDD* major depressive disorder.

^a^Analyzed by the Chi-square test.

^b^Analyzed by Mann–Whitney U test.

^c^Independent sample *t*-tests.

### Serum lipids findings

**Fig. 1:**
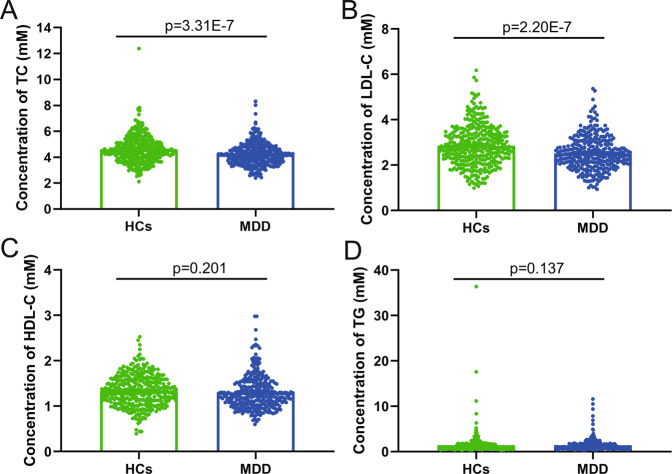
Serum lipids levels in healthy controls (HCs) and major depressive disorder (MDD) patients. **A**–**D** Comparison of serum total cholesterol (TC; **A**), low-density lipoprotein cholesterol (LDL-C; **B**), high-density lipoprotein cholesterol (HDL-C; **C**) and triglyceride (TG; **D**) levels between the two groups. Data are presented as mean ± S.E.M.

The concentrations of serum TC, LDL-C, HDL-C, and TG from subjects in sample set 1 were presented in Fig. 1. Compared to HCs, MDD patients had significantly lower concentrations of TC (*p* = 3.31E−7) and LDL-C (*p* = 2.20E−7), yet HDL-C (*p* = 0.201) and TG (*p* = 0.137) levels were similar between MDD patients and HCs. And LDL-C concentration in serum was found to be significantly positively correlated with TC level (*r* = 0.8471, *p* = 1.23E−186; Fig. S1). When comparing analysis with additional age distribution in HCs, DN-MDD, and DT-MDD groups (Table S3), the results regarding TC and LDL-C concentrations remained significant, and HDL-C and TG levels still showed no significant differences between the groups, though there was a significant difference in HDL-C concentration between the 36–55 age subgroup (*p* = 0.003). Furthermore, TC and LDL-C concentrations were higher in the DT-MDD group relative to the DN-MDD group, especially in the 18–35 age subgroup (TC, *p* = 0.012; LDL-C, *p* = 0.014).

### IPA analysis

**Fig. 2:**
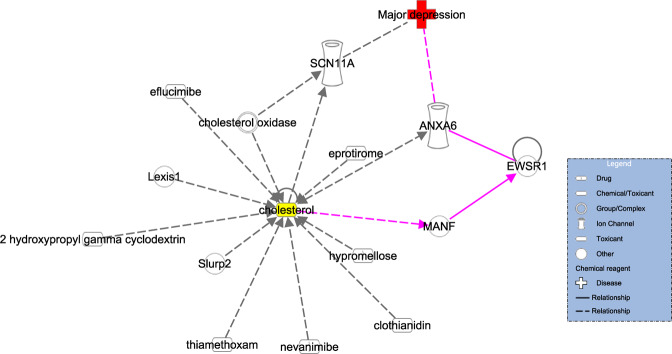
Potential networks among cholesterol, MANF, and major depressive disorder. Ingenuity Pathway Analysis database indicates that the MANF/EWSR1/ANXA6 pathway might act as the connecting bridge between cholesterol and major depressive disorder.

To investigate the role of lipid regulator MANF in depression, the IPA database was used to explore the potential network of associations among cholesterol, MANF, and MDD. Analysis of the obtained molecular network showed that cholesterol might be involved in the pathogenesis of MDD via regulating the MANF/EWSR1/ANXA6 pathway (Fig. 2).

### Correlations between MANF/EWSR1/ANXA6 pathway and MDD

**Fig. 3:**
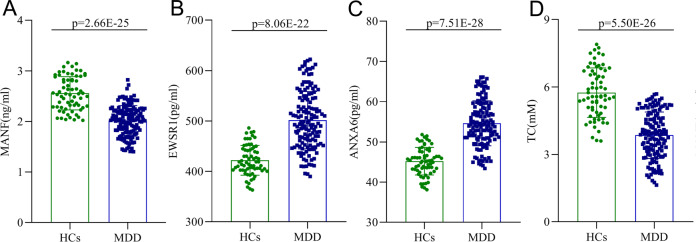
Levels of MANF, EWSR1, ANXA6, and TC in healthy controls (HCs) and major depressive disorder (MDD) groups. **A**–**C** Concentrations of MANF (**A**), EWSR1 (**B**), and ANXA6 (**C**) between the two groups. **D** The concentration of TC between the two groups. Data are presented as mean ± S.E.M.

Given the hypothesis that cholesterol might be correlated with MDD via MANF/EWSR1/ANXA6 pathway, we further detected the levels of these molecules in the serum of subjects in sample set 2. As shown in Fig. 3A–C, compared to HCs, MDD patients had significantly lower level of MANF (*p* = 2.66E−25) and higher levels of EWSR1 (*p* = 8.06E−22) and ANXA6 (*p* = 7.51E−28). Correlation analysis showed that MANF level (*r* = −0.532, *p* = 1.03E−16) was significantly negatively correlated with HDRS score, and both EWSR1 level (*r* = 0.480, *p* = 1.62E−13) and ANXA6 level (*r* = 0.538, *p* = 3.69E−17) were significantly positively correlated with HDRS score (Fig. S2). Meanwhile, we found that compared to DN-MDD patients, DT-MDD patients had significantly higher levels of MANF (*p* = 1.95E−12), while lower levels of EWSR1 (*p* = 4.33E−09) and ANXA6 (*p* = 2.85E−17) (Fig. S3). These results demonstrated that MANF/EWSR1/ANXA6 pathway was significantly affected in MDD patients, and the disturbed levels of these molecules were significantly improved after treatment.

### Correlations between MANF/EWSR1/ANXA6 pathway and hypolipidemia

In sample set 2, we also detected the level of TC in MDD patients and HCs. The results further confirmed that there was a significantly lower level of TC in MDD patients than in HCs (*p* = 5.50E−26; Fig. 3D). Moreover, we found that DT-MDD patients had a significantly higher level of TC compared to DN-MDD patients (*p* = 4.22E−28; Fig. S4). Thus, results from the sample set 1 and set 2 consistently demonstrated that there was hypolipidemia in MDD patients, and it was improved after treating with antidepressant drugs. Meanwhile, correlation analysis showed that the levels of MANF (*r* = 0.554, *p* = 2.91E−18), EWSR1 (*r* = −0.582, *p* = 2.12E−20) and ANXA6 (*r* = −0.630, *p* = 1.26E−24) were significantly correlated with TC level (Fig. S5). The present findings suggested that there were close relationships between MANF/EWSR1/ANXA6 pathway and hypolipidemia in MDD patients.

### MANF/EWSR1/ANXA6 as biomarkers for MDD

**Fig. 4:**
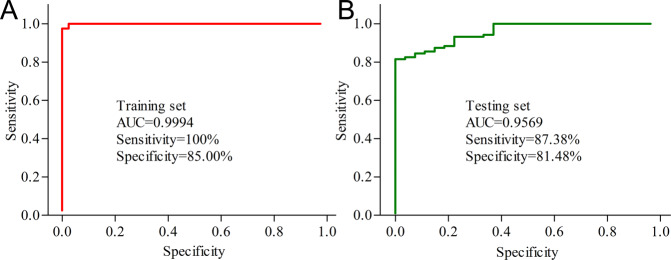
Diagnostic performance of the obtained discriminative model. **A**, **B** A discriminative model consisting of MANF, EWSR1, and ANXA6 discriminated MDD subjects from healthy controls with an area under the receiver operating characteristic curve (AUC) of 0.9994 in the training set (**A**) and 0.9569 in the testing set (**B**).

The subjects in sample set 2 were divided into training set and testing set. Using logistical regression analysis, we obtained a discriminative model consisting of MANF, EWSR1 and ANXA6, which could effectively separate MDD patients from HCs. This discriminative model was as follows: p(y = 1)=1/(1 + exp(5.263*MANF − 0.028*EWSR1 − 0.368*ANXA6 + 17.506)). Then, we used ROC curve analysis to assess its diagnostic performance. The results showed that this discriminative model yielded an AUC of 0.9994 in the training set (sensitivity = 100%, specificity = 85.00%; Fig. 4A) and 0.9569 in the testing set (sensitivity = 87.38%, specificity = 81.48%; Fig. 4B). These results suggested that this discriminative model could be an ‘excellent’ classifier of HCs and MDD patients, and these molecules held the promise as the potential biomarkers for diagnosing MDD.

## Discussion

Our study found that the serum lipids concentrations, especially TC and LDL-C, were significantly lower in MDD patients than in HCs. No significant differences were found in HDL-C and TG levels between the two groups. Moreover, based on the IPA database analysis, we hypothesized that MANF/EWSR1/ANXA6 pathway might act as a bridge between low serum lipids and depression. Further investigation showed that the serum levels of MANF, EWSR1, and ANXA6 were significantly disturbed in MDD patients. And a discriminative model consisting of MANF, EWSR1, and ANXA6 was identified, which enabled the discrimination of MDD subjects from HCs with AUCs of 0.9994 in the training set. Moreover, in testing set, HCs and MDD patients were not completely sex-matched, the discriminative model could still yield an AUC of 0.9569 in testing set, highlighting the diagnostic robustness of this discriminative model. These results indicated that MANF/EWSR1/ANXA6 pathway might have an important role in the pathogenesis of depression.

The lower levels of lipids in MDD patients here were in line with some previous studies [30–32], but contradicted with others [33], even some previous epidemiological studies found that there was no difference in serum lipid levels between the groups [34, 35]. Besides, it has been stressed that the relationship between brain levels and serum levels is quantified, while there is evidence from rodent studies that peripheral and brain cholesterol may be independently regulated [36]. In the current study with a large number of subjects, we found that the levels of TC and LDL-C were significantly decreased in MDD patients. Moreover, considering the potential effects of age on serum lipids, we further divided the included subjects into three groups according to the different age ranges, and obtained similar results. Our results further identified the change of serum lipids in depression, and could partially explain why the previous studies reported contradictory results. Noteworthy, anti-depression drug treatment can significantly increase the concentrations of TC and LDL-C, especially in the 18–35 age subjects. Paroxetine and sertraline, two effective and widely used drugs for MDD, have negative effects on the serum levels of TC and LDL-C [37, 38]. Therefore, our results could also be used as evidence to support citalopram, not paroxetine and sertraline, to be a treatment of choice for patients with depression affected by dyslipidemia [37]. In addition, the effect of antidepressants in improving dyslipidemia may be age-limited and primarily occurs in the young adult population, which warrants further investigation.

MANF levels are known to be disturbed in neurodegenerative diseases [39] or ischemic stroke [40]. So far, to our knowledge, this was the first study that investigated the concentration of serum MANF in MDD patients. Here, we found that the serum MANF level was significantly decreased in MDD patients, and significantly correlated to depression severity (negatively) and TC level (positively). Meanwhile, both EWSR1 and ANXA6 were found to be significantly increased in MDD patients, and significantly correlated to depression severity (positively) and TC level (negatively). EWSR1 is ubiquitously expressed in most cell types, which involves all tissues and almost all cell types, and it has been reported that involves in various cellular processes and organ development [41]. Several studies have shown that EWSR1 is associated with central nervous system disorders, such as amyotrophic lateral sclerosis [42] and frontotemporal dementia [43]. ANXA6 belongs to a family of membrane-binding proteins that are Ca^2+^-dependent [44]. Previous studies revealed that ANXA6 is linked to its ability to bind phospholipids in cellular membranes dynamically and reversibly, particularly during the regulation of exocytic and endocytic pathways [45, 46]. It is independent of neuronal activity and resistant to detergent extraction consistent with an interaction with cytoskeletal proteins [47]. These results suggest that MANF, EWSR1, and ANXA6 might play vital roles in the pathogenesis of depression. Besides, in this study, antidepressant treatment significantly improved the disturbed levels of MANF/EWSR1/ANXA6 pathway and TC. The MANF and EWSR1 genes were associated with the regulation of lipid [48], and ANXA6 has been shown to reside in lipid rafts and localized in the axon initial segment during neuronal development [49]. Thus, the effect of antidepressants in improving dyslipidemia may be mediated by the MANF/EWSR1/ANXA6 pathway, which is worthy to be investigated in the future.

In addition, to find out the potential biomarkers that could truly reflect the pathophysiologic changes inherent in depression state, only DN-MDD patients were assigned to the training set. However, considering that antidepressant drugs were commonly used in clinical practice, then testing set including unselected MDD patients (both DN-MDD and DT-MDD) was used here to independently assess the diagnostic generalizability of the identified potential biomarkers. This method has been successfully conducted in our previous studies [50, 51]. In this study, we found that the discriminative model consisting of MANF, EWSR1, and ANXA6 could still yield an AUC of 0.9569 in testing set, highlighting the diagnostic robustness of this discriminative model. Meanwhile, considering the relative stability of MANF, EWSR1, and ANXA6 in blood and the ability to be rapidly and inexpensively measured, these molecules could be potential biomarkers for MDD. Taken together, our results established that MANF/EWSR1/ANXA6 pathway might as the bridge between hypolipidemia and MDD.

There were few reports about the relation of MANF/EWSR1/ANXA6 pathway to hypolipidemia-associated MDD. Our findings would provide a new direction for further investigating this relationship. Besides, MANF, EWSR1, and ANXA6 were found to be significantly altered in the serum of MDD patients and correlated with serum lipid and the severity of depression symptoms. These molecules might serve as additional state or trait biomarkers in depression. Our results also indicated a possible role of these molecules in the pathogenesis or progression of MDD, which could broaden our knowledge on the possible involvement of lipid metabolism in MDD. However, it is still unclear if hypolipidemia is the cause of depression or it occurs as a secondary effect in MDD patients. Thus, longitudinal studies involving serum lipid examination and MANF/EWSR1/ANXA6 pathway assessment are needed to further explain the possible role of hypolipidemia in the pathogenesis of MDD.

Several limitations of this study should be mentioned here. First, the present study about serum lipid examination was retrospective, open, and uncontrolled design, although a large number of MDD patients were enrolled. Second, the effects of other influencing factors in this study, such as dietary habits or psychiatric comorbidity cannot be excluded [52–54]. Third, we only detected MANF/EWSR1/ANXA6 pathway in serum, future studies should directly detect the levels of these molecules in the central nervous system, such as cerebrospinal fluid, to further pinpoint the role of this pathway in depression.

## Conclusion

Our study first measured the MANF/EWSR1/ANXA6 pathway in the serum of MDD patients. In conclusion, we found that compared to HCs, MDD patients had significantly lower levels of TC, LDL-C, and MANF, and higher levels of EWSR1 and ANXA6. Meanwhile, MANF, EWSR1, and ANXA6 were significantly correlated to TC concentrations and depression severity, and they could be potential biomarkers for diagnosing MDD. These results suggested that MANF/EWSR1/ANXA6 pathway might act as the bridge between hypolipidemia and depression. Our findings would be helpful for the future development of objective diagnostic methods for MDD and provide novel insights into exploring the pathogenesis of depression.

## Supplementary information

Supplemental Information
