# Supplementary material for: MANF/EWSR1/ANXA6 pathway might as the bridge between hypolipidemia and major depressive disorder
Source: Transl Psychiatry. 2022 Dec 30;12:527. doi: 10.1038/s41398-022-02287-0 (PMC9803680; doi:10.1038/s41398-022-02287-0)
Supplement: Supplementary file 1 — Supplemental Information [file 41398_2022_2287_MOESM1_ESM.docx]

**MANF/EWSR1/ANXA6 pathway might as the bridge between** **hypolipidemia and major depressive** **disorder**

***Supplemental Information***

**Supplementary tables and figures**

**Table S1 Characterization of the major depressive patient subjects in sample set 1.**

| Characteristics | DN-MDD (N=130) | DT-MDD (N=224) |
| --- | --- | --- |
| Marital status  Single  Married  Divorced  Widowed  Levels of education  Low  Middle  High  Work situation  Unemployed  Others not working  Working  In training  Retired  Housewife/-man  Personal history  Drinking  Never  Moderate  Heavy  Smoking  Never  Moderate  Heavy  Types of antidepressants (n, %)  Fluoxetine  Sertraline  Escitalopram  Olanzapine  Mirtazapine  [Venlafaxine](javascript:;)  Others | 49 (37.69%)  74 (56.92%)  4 (3.08%)  3 (2.31%)  41 (31.54%)  44 (33.85%)  45 (34.62%)  10 (7.69%)  18 (13.85%)  68 (52.31%)  6 (4.62%)  18 (13.85%)  10 (7.69%)  114 (87.69%)  13 (10.00%)  3 (2.31%)  110 (84.62%)  13 (10.00%)  7 (5.38%)  NA  NA  NA  NA  NA  NA  NA | 83 (37.05%)  120 (53.57%)  16 (7.14%)  5 (2.23%)  74 (33.04%)  91 (40.63%)  59 (26.34%)  26 (11.61%)  17 (7.59%)  116 (51.79%)  18 (8.04%)  23 (10.27%)  24 (10.71%)  182 (81.25%)  32 (14.29%)  10 (4.46%)  188 (83.93%)  30 (13.39%)  6 (2.68%)  *Total number =* 325*^#^*  31 (9.54%)  36 (11.08%)  50 (15.38%)  77 (23.69%)  38 (11.69%)  49 (15.08%)  44 (13.54%) |

Highest achieved educational level was determined and defined in three groups for analysis: low level of education (medium-level secondary education or below), medium level of education (higher-level secondary education or vocational education) and high level of education (diploma level or university education).

^#^ Some patients used more than one antidepressant.

**Table S2 Characterization of the major depressive patient subjects in sample set 2.**

| Characteristics | DN-MDD (N=75) | DT-MDD (N=68) |
| --- | --- | --- |
| Marital status  Single  Married  Divorced  Widowed  Levels of education  Low  Middle  High  Work situation  Unemployed  Others not working  Working  In training  Retired  Housewife/-man  Personal history  Drinking  Never  Moderate  Heavy  Smoking  Never  Moderate  Heavy  Types of antidepressants (n, %)  Fluoxetine  Sertraline  Escitalopram  Olanzapine  Mirtazapine  [Venlafaxine](javascript:;)  Others | 28 (37.33%)  41 (54.67%)  5 (6.67%)  1 (1.33%)  20 (26.67%)  21 (28.00%)  34 (45.33%)  11 (14.67%)  6 (8.00%)  41 (54.67%)  6 (8.00%)  3 (4.00%)  8 (10.67%)  58 (77.33%)  12 (16.00%)  5 (6.67%)  62 (82.67%)  11 (14.67%)  2 (2.67%)  NA  NA  NA  NA  NA  NA  NA | 22 (32.35%)  41 (60.29%)  4 (5.88%)  1 (1.47%)  16 (23.53%)  23 (33.82%)  29 (42.65%)  7 (10.29%)  6 (8.82%)  40 (58.82%)  4 (5.88%)  6 (8.82%)  5 (7.35%)  53 (77.94%)  7 (10.29%)  8 (11.76%)  54 (79.41%)  9 (13.24%)  5 (7.35%)  *Total number =* 126*^#^*  17 (13.49%)  12 (9.52%)  15 (11.90%)  32 (25.40%)  11 (8.73%)  20 (15.87%)  19 (15.08%) |

Highest achieved educational level was determined and defined in three groups for analysis: low level of education (medium-level secondary education or below), medium level of education (higher-level secondary education or vocational education) and high level of education (diploma level or university education).

^#^ Some patients used more than one antidepressant.

**Table S3** **Serum TC, LDL-C, HDL-C and TG concentrations in sample set 1.**

| Age  subgroups |  | TC |  |  |  |  | LDL-C |  |  |
| --- | --- | --- | --- | --- | --- | --- | --- | --- | --- |
|  | HCs | DN-MDD | DT-MDD | p value |  | HCs | DN-MDD | DT-MDD | p value |
| Total  18-35 (y)  36-55 (y)  56-70 (y) | 4.60±0.06  (360)  4.28±0.08  (163)  4.81±0.08  (152)  5.03±0.15  (45) | 4.12±0.07  (130)  3.86±0.10  (65)  4.23±0.16  (31)  4.45±0.12  (34) | 4.28 ±0.07  (224)  4.24±0.10  (104)  4.26±0.12  (63)  4.35±0.12  (57) | 1.01E-6  0.004  1.97E-4  0.003 |  | 2.85±0.05  (360)  2.56±0.06  (163)  3.00±0.08  (152)  3.36±0.14  (45) | 2.45±0.06  (130)  2.32±0.10  (65)  2.52±0.15  (31)  2.62±0.10  (34) | 2.56±0.05  (224)  2.59±0.08  (104)  2.51±0.11  (63)  2.55±0.11  (57) | 1.38E-6  0.031  3.83E-4  1.39E-6 |

| Age  subgroups |  | HDL-C |  |  |  |  | TG |  |  |
| --- | --- | --- | --- | --- | --- | --- | --- | --- | --- |
|  | HCs | DN-MDD | DT-MDD | p value |  | HCs | DN-MDD | DT-MDD | p value |
| Total  18-35 (y)  36-55 (y)  56-70 (y) | 1.34±0.02  (360)  1.35±0.03  (163)  1.34±0.03  (152)  1.32±0.05  (45) | 1.35±0.04  (130)  1.31±0.04  (65)  1.29±0.07  (31)  1.47±0.09  (34) | 1.28±0.03  (224)  1.31±0.04  (104)  1.16±0.04  (63)  1.33±0.05  (57) | 0.113  0.331  0.003  0.403 |  | 1.43±0.12  (360)  1.34±0.23  (163)  1.58±0.15  (152)  1.24±0.08  (45) | 1.28±0.07  (130)  1.00±0.07  (65)  1.65±0.19  (31)  1.39±0.14  (37) | 1.50±0.10  (224)  1.24±0.13  （104）  1.91±0.29  (63)  1.56±0.11  (57) | 0.172  0.630  0.291  0.100 |

*DN-MDD*, drug-naïve major depressive disorder; *DT-MDD*, drug-treatment major depressive disorder; *HCs*, healthy controls; *HDL-C*, high-density lipoprotein cholesterol; *LDL-C*, low-density lipoprotein cholesterol; *TC*, total cholesterol; *TG*, triglyceride. Data are presented as mean ± S.E.M.


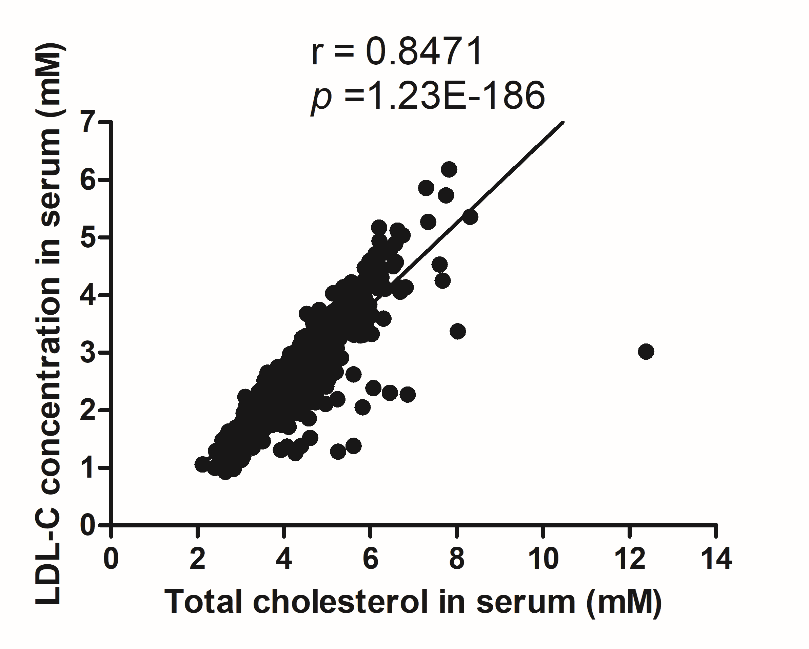


**Fig S1. Total cholesterol level is positively correlated with the concentration of low-density lipoprotein cholesterol (LDL-C) in serum.**

**
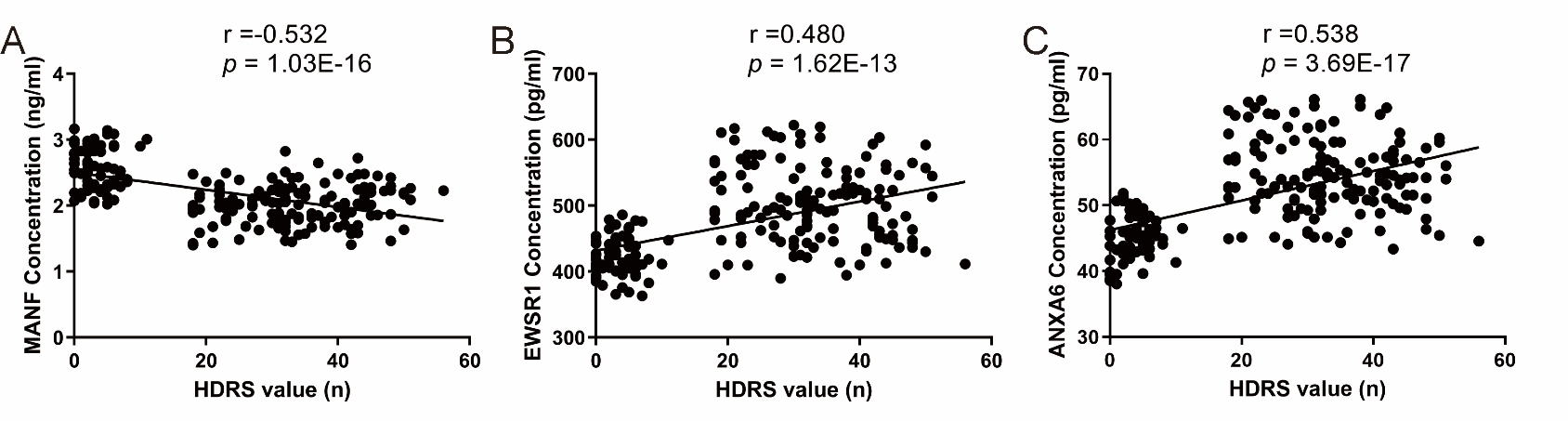
**

**Fig S2. Pearson correlation between MANF, EWSR1 and ANXA6 concentrations and HDRS value. (A-C)** MANF level (A) was significantly negatively correlated with HDRS score, and both EWSR1 level (B) and ANXA6 level (C) were significantly positively correlated with HDRS score.


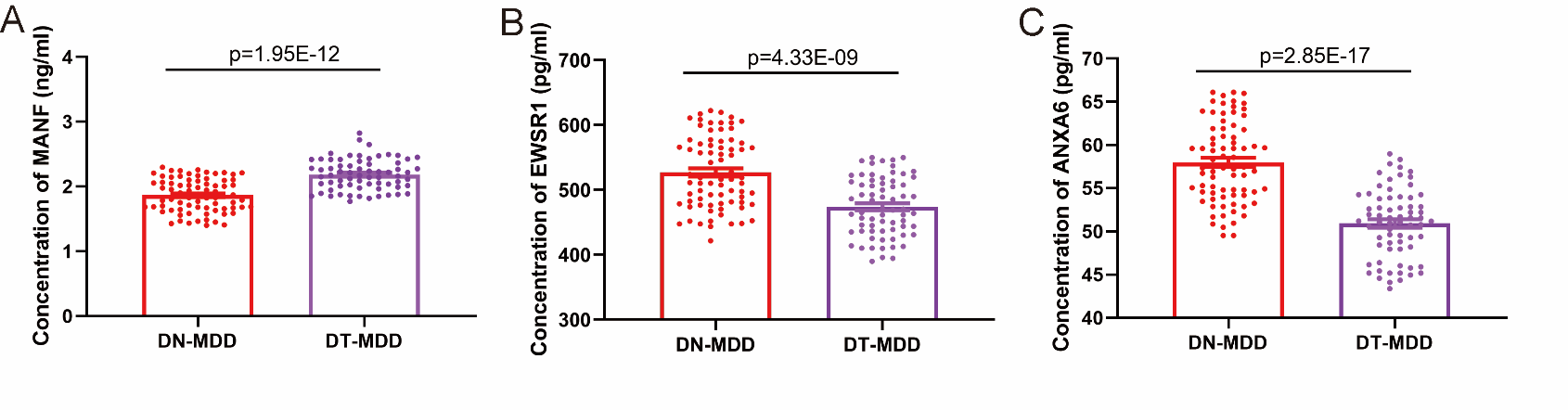
**Fig S3. Concentrations of MANF, EWSR1 and ANXA6 in DN-MDD and DT-MDD groups. (A-C)** Compared to DN-MDD patients, DT-MDD patients had significantly higher level of MANF (A) and lower levels of EWSR1 (B) and ANXA6 (C). Data are presented as mean ± S.E.M.


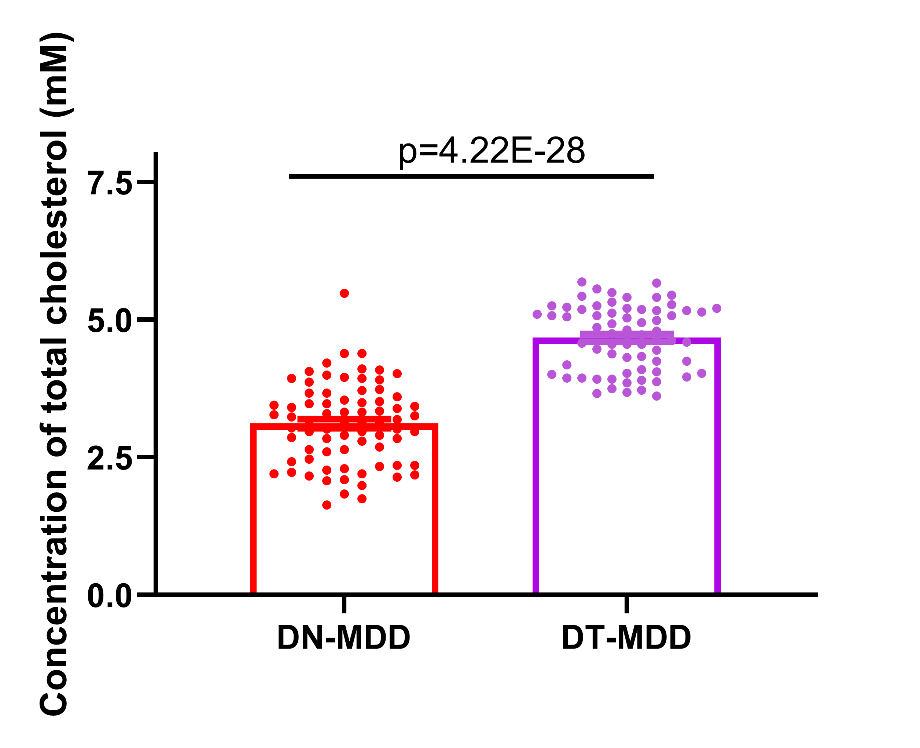


**Fig S4. Concentrations of total cholesterol level (TC) in DN-MDD and DT-MDD groups.** **Data are presented as mean ± S.E.M.**


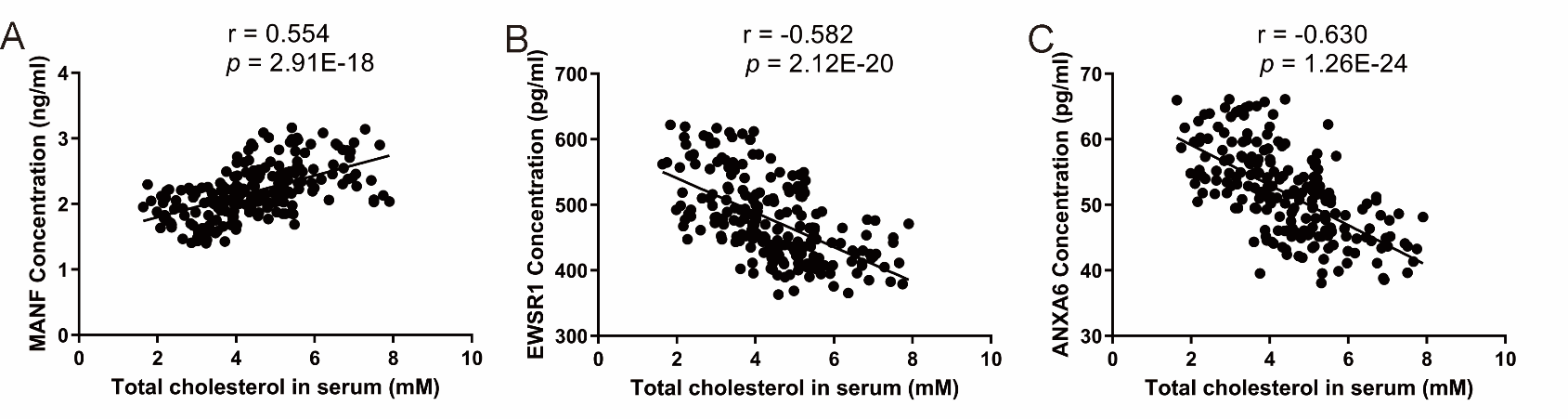


**Fig S5. Pearson correlation between MANF, EWSR1 and ANXA6 concentrations and TC** **level. (A-C)** MANF level (A) was significantly positively correlated with TC level, and both EWSR1 level (B) and ANXA6 level (C) were significantly negatively correlated with TC level.
